# Antinuclear antibodies and their detection methods in diagnosis of connective tissue diseases: a journey revisited

**DOI:** 10.1186/1746-1596-4-1

**Published:** 2009-01-02

**Authors:** Yashwant Kumar, Alka Bhatia, Ranjana Walker Minz

**Affiliations:** 1 Department of Pathology and Laboratory Medicine, Grecian Superspeciality, Heart, Cancer and Multispeciality Hospital, Sector 69, Mohali, India; 2 Department of Experimental Medicine and Biotechnology, Post Graduate Institute of Medial Education and Research, Chandigarh, India; 3 Department of Immunopathology, Post Graduate Institute of Medial Education and Research, Chandigarh, India

## Abstract

It has been more than 50 years since antinuclear antibodies were first discovered and found to be associated with connective tissue diseases. Since then different methods have been described and used for their detection or confirmation. For many decades immunofluorescent antinuclear antibody test has been the "gold standard" in the diagnosis of these disorders. However to increase the sensitivity and specificity of antinuclear antibody detection further approaches were explored. Today a battery of newer techniques are available some of which are now considered better and are competing with the older methods. This article provides an overview on advancement in antinuclear antibody detection methods, their future prospects, advantages, disadvantages and guidelines for use of these tests.

## Review

Connective tissue diseases (CTD) are a group of autoimmune disorders which are characterized by presence of antinuclear antibodies (ANA) in the blood of patients. ANA are a specific class of autoantibodies that have the capability of binding and destroying certain structures within the nucleus of the cells [1]. Although lower amounts of these antibodies can be seen in the normal population as well, a spurt in titers is seen in patients of CTD. Not only are these antibodies involved in the disease pathogenesis, but they also constitute the basis for diagnosis and treatment of CTD. Their detection with high sensitivity and specificity is therefore of utmost importance. Various detection methods are in use and there is continuous pouring of newer techniques to facilitate diagnosis and therapeutic monitoring in CTD patients. In this review we have discussed in brief how ANA were discovered and found to be associated with CTD. This article also gives an overview on advancement in various ANA detection methods, their future prospects along with advantages, disadvantages and guidelines for use of these tests.

## Historical aspects of ANA

In 1941, Klemperer, Pollack and Baehr first described systemic lupus erythematosus (SLE) as one of the CTD [2]. Then in 1948 Malcom Hargrave, Helen Richmond and the medical resident Robert Morton noted the presence of previously unknown cells in the bone marrow of a patient with SLE. They called these LE cells and described them as mature polymorphonuclear leukocytes which had phagocytosed the liberated nuclear material of another leukocyte [3]. This extremely important discovery laid the foundation of research for ANA. Since then, ANA has been divided into specific subtypes based on the nuclear or cytoplasmic component they attack i.e. anti-DNA, anti-histone etc.

## ANA – the two broad subtypes

Presently the ANA have been categorized in to 2 main groups:

### Autoantibodies to DNA and histones

These include antibodies against single and double-stranded DNA (dsDNA) discovered way back in 1957. Significant levels of anti-dsDNA antibodies are considered to be confirmatory in diagnosis of SLE. This was followed by detection of anti-histone antibodies in 1971 which are indicative of drug-induced SLE [4-8].

### Autoantibodies to extractable nuclear antigens (ENA)

Besides DNA and histones, autoantibodies may also target other nuclear antigens. These nuclear antigens were named ENA as originally they were extracted from the nuclei with saline [8]. Autoantibody to Smith antigen (Sm) which is considered to be specific for SLE was the first anti-ENA detected in 1966 [9]. Thereafter further subtypes of ENA i.e. ribonucleoproteins (RNP), SSA/Ro, or SSB/La, Scl-70, Jo-1 and PM1 were more clearly identified [10-17]. Although most of these ENA are disease specific, still a significant overlap exists. The sensitivity and specificity also varies depending upon the type of underlying CTD. A list of clinically important ANA with their sensitivity and specificity of identifying an autoimmune disorder can be seen in table 1[18,19].

**Table 1:** Sensitivity and specificity of ANA and its clinically important subtypes [18,19]

**Autoantibodies**	**Associated CTD**	**Sensitivity**	**Specificity**
**ANA**	SLE	93	57
	Sjogren's syndrome	48	52
	SS	85	54
	PM/dermatomyositis	61	63
	Raynaud phenomena	64	41

**Specific ANA**			
Anti-dsDNA	SLE	57	97
Anti-Sm	SLE	25–30	High*
Anti-SSA/Ro	Sjogren's syndrome, subacute cutaneous SLE, Neonatal lupus syndrome	8–70	87
Anti-SSB/La	Sjogren's syndrome, subacute cutaneous SLE, Neonatal lupus syndrome	16–40	94
Anti-U3-RNP	SS	12	96
Anticentromere	Limited cutaneous SS	65	99.9
Scl-70	SS	20	100
Jo-1	PM	30	95

* Precise data not available.

In the last few years many other autoantibodies like topoisomerase-I (Topo-I), centromere protein (CENP)-B, RNA-polymerase I-III (RNA-pol I-III), MU, TM, Ku, Mi-2, RA33 etc. have also been described. While of scientific interest, typing of many of these antibodies has not found its way in to the clinical practice. Certain autoantibodies against cytoplasmic and cell membrane components though present are less relevant in diagnostics [20,21].

## Techniques for ANA detection

Presence of autoantibodies in the sera of the patient constitutes one of the criteria used for diagnosis of CTD (table 2). Besides clinical diagnosis the ANA subtyping also helps in identifying a specific CTD [22]. Although a battery of laboratory tests are available for ANA detection indirect immunofluorescence antinuclear antibody test (IF-ANA) and enzyme immunoassay (EIA)/enzyme linked immunosorbent assay (ELISA) are commonly used in day to day practice. Some of them are considered outdated while others like flowcytometry and recently introduced nanotechnology involving antigen arrays are still in experimental stages.

**Table 2:** Significance of positive ANA test in CTD and some non-autoimmune conditions [36]

**Useful for diagnosis**	**Useful for monitoring or prognosis**
1) Lupus erythmatosus (LE)	1) Juvenile chronic oligoarticular arthritis
SLE	2) Raynaud phenomenon
	
Discoid LE	**Not useful for diagnosis**
	
Subacute cutaneous LE	
Neonatal LE	1) Relatives of patients with CTD
Overlap of two or more LE subsets	2) Other autoimmune diseases (e.g., Rheumatoid arthritis, Idiopathic thrombocytopenia purpura, primary biliary cirrhosis, autoimmune thyroiditis)
Overlap of LE with other CTD	3) Drugs (e.g., procainamide, hydralazine)
2) SS	4) Silicone breast implant patients
Cutaneous SS (morphea)	5) Fibromyalgia
Systemic SS	6) Chronic infections
a) Limited disease	7) Neoplasms
b) Diffuse disease	8) Elderly persons
3) PM/Dermatomyositis	9) Pregnant women
4) Sjögren's syndrome (primary and secondary)	10) Healthy persons
5) Mixed CTD	
6) Overlap and undifferentiated CTD	

### IF-ANA: The standard ANA testing technique

Before development of IF-ANA test, LE cell preparation was the only method used for diagnosis of SLE. IF-ANA was designed by George Friou in 1957 [23]. Since then it has been the most widely used test for diagnosis of CTD. It is inexpensive and easy to perform, with high sensitivity and specificity [24]. The test detects the presence of ANA in the blood of the patient which adhere to reagent test cells (substrate), forming distinct fluorescence patterns that are associated with certain autoimmune diseases. Initially different substrates like tissue sections, desquamated cells, chicken erythrocytes and HeLa cells were tried but later on tissue sections using rat liver or a composite multiblock substrate (mouse stomach, rat liver and kidney) became the standard substrate. In 1975 HEp-2 cells were introduced which have further increased the sensitivity of the test. These are the cultured cells of laryngeal squamous cell carcinoma and are available commercially in the form of prefixed on glass slides. Majority of the laboratories around the world are now using HEp-2 cell substrates [25].

The correct interpretation of the IF-ANA results is important and must always be correlated with the patient's symptoms and signs. While reporting IF-ANA three parameters are evaluated; these include the pattern of fluorescence, substrate used and the titer of a positive test. A negative IF-ANA result essentially excludes possibility of active CTD.

### Fluorescence patterns and intensity

**Figure 1:**
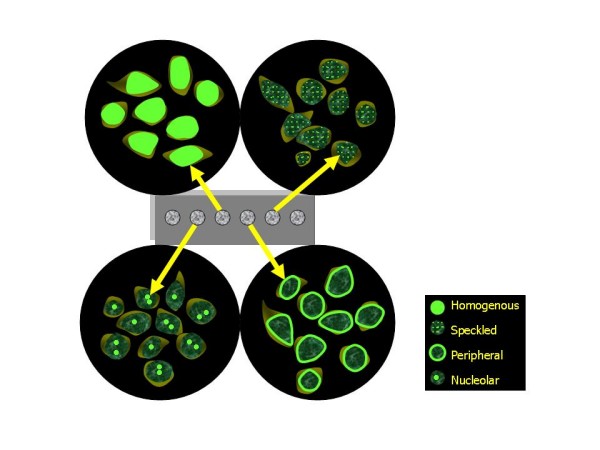
**Diagrammatic representation of common nuclear patterns observed under fluorescence microscopy**.

Different staining patterns are reported which give clues as to the significance of the ANA and type of CTD (table 3, figure 1):

**Table 3:** Common IF-ANA patterns associated with specific diseases

**ANA pattern**	**Antigen**	**Associated diseases**
Speckled	ENA, RNP, Sm, SSA/Ro, SSB/La, Scl-70, Jo-1, ribosomal-P	SLE, Mixed CTD, SS, Primary Sjogren's syndrome, PM
Homogenous	dsDNA, Histones	SLE, Drug induced SLE
Peripheral (rim)	RNP, Sm, SSA/Ro	SLE, SS
Nucleolar	Anti-PM-Scl, anti-RNA polymerase I-III, anti-U3-RNP, To RNP	SS, PM
Centromere	CENP A-E	Limited SS

1. Nuclear patterns: homogeneous, speckled (fine and coarse), peripheral/rim, nucleolar, centromeric, PCNA (proliferating cell nuclear antigen), nuclear dots, nuclear membrane, diffuse grainy.

2. Cytoplasmic patterns: speckled, mitochondrial-like, ribosomal-like, Golgi apparatus, lysosomal-like, cytoskeletal filaments (actin, vimentin, cytokeratin)

3. Mitotic patterns: mitotic spindle, centrosomes, NuMA (nuclear mitotic apparatus), midbody, CENP-F (centromere protein)

Among these homogenous, speckled, peripheral and nucleolar patterns are more commonly observed and of clinical importance. With any of these fluorescence patterns intensity of staining with a qualitative scale of values from + to ++++ should also be reported as fluorescence intensity is generally proportional to antibody concentration and predicts the severity of the CTD.

### ANA substrate

Sera of some patients with SLE may be negative on animal substrates i.e. mouse kidney or rat liver but are positive on human substrate i.e. Hep-2 cell lines [26-28]. Due to variable sensitivity with the substrate used it is essential to report the type of substrate being used by the lab.

### ANA titer

It is directly proportional to antibody concentration and expressed with a quantitative scale of values. Its evaluation is crucial as low titer is less significant than a high titer and may be seen even in healthy individuals. There are many studies which have attempted to determine the optimum screening dilution of sera for ANA testing. A titer of 1:160 is taken as significant for the diagnosis of CTDs in majority of laboratories [29,30].

Although IF-ANA test is widely used and considered to be gold standard still the results may sometimes be misinterpreted. As it detects several different antibodies cross-reactions can occur. In up to 3% of the normal population it can give false positive result. Also ANA levels tend to rise when symptoms flare and fall, often being undetectable, when symptoms are mild or patient is in remission. Moreover each CTD has specific antibody associated with it and sometimes it is difficult to specify or categorize an autoantibody [31,32]. Certain patterns i.e. nucleolar and centromeric are less well defined by IF-ANA tests. The test therefore is mainly used for screening rather than to diagnose a CTD.

### EIA/ELISA

There are two types of EIA or ELISA methods currently used for ANA testing. One is called generic assay which detects ANA of broad specificity similar to IF-ANA and other is antigen specific assay that detects ANA and reacts with a single autoantigen i.e. dsDNA, SS-A/Ro, SS-B/La, Scl-70, Sm, Sm/RNP etc. In antigen specific assay multiple antigens are coated on to microtitre plates, usually a combination of SSA/Ro, SSB/La, Sm, and U1-RNP, with many also including Jo-1 and Scl70. This new test is both highly specific and sensitive and substantially decreases the time involved when screening large numbers of patient samples. The test is simple to perform, can be automated and does not require highly trained operators who can recognize microscopic patterns. The EIA/ELISA is therefore becoming the most widely used method not only for routine screening but also for detection of specific ANA. Kits available in the market either utilize extracts of tissue containing various nuclear components or molecules synthesized by recombinant technology. The later may include individual recombinant molecules such as SS-A/Ro, or combinations of molecules which increase the sensitivity of the test. In a recent study, the performance of ELISA test was compared with the "gold standard" IF-ANA test. The agreement that a serum is ANA positive was 87% to 95% when comparing the ELISA and IF-ANA test results [33]. The sensitivity of the various ELISAs was 69% to 98% and the specificity ranged between 81% and 98%. These figures were arrived at using sera that were positive at 1:160 by the IF-ANA test. The above comparison figures were much lower for sera with IF-ANA titer of 1:40.

Although the second multicentre European study showed that ELISA methods are improving [34], the recent study by Bizzaro et al suggests that the problem of false positive results in ELISA is still widespread [35]. ELISA may miss anti-SSA/Ro even when using the reference sera. This is probably a result of the vigorous antigen preparation methods. Sera that react only with conformational antigens can also miss the presence of antigen. The ELISA techniques have also been found to miss a low titer positive ANA as well as sera with specific ANA. Presently, ELISA tests therefore may be adequate to screen sera only with intermediate to high titers. It remains to be seen from further studies whether the performance of screening ANA tests by ELISA would match that by the fluorescent technique [36].

### Techniques used for detection of specific ANA

#### Detection of antibodies against dsDNA

Three techniques are currently in use for the detection of anti-dsDNA antibodies:

1) IF-ANA test using *Crithidia luciliae *as the substrate (CLIF)

2) The Farr assay

3) ELISA dsDNA

#### CLIF

Aarden and his colleagues in 1975 used IF-ANA test for detection of dsDNA antibodies by using a haemoflagellate C. luciliae as the substrate [37]. The organism is related to trypanosomes and is equipped with an intracellular organelle, the kinetoplast. The kinetoplast contains dsDNA in high concentration while apparently not containing any other recognizable nuclear antigens. The test is most useful in primary diagnosis of SLE with high specificity when compared to ELISA [38].

Although the sensitivity is comparable to Farr assay, CLIF is easy to perform, possesses an intrinsic check on the immunoglobulin character of the DNA-binding activity, determines the Ig classes and subclasses of antibodies to DNA. In addition, there is an absence of interference with antibodies to single-stranded DNA [39].

#### Farr assay

The Farr assay is a radio-labeled assay which quantifies antibody to a given antigen in sera through precipitation of antibody-antigen complexes on addition of ammonium sulfate at high concentration. A radio-labeled antigen (dsDNA) allows the quick determination of proportion of the antibody in the precipitate. The Farr assay is quite specific and has been advocated as the most reliable assay. However, it is time-consuming, technically difficult and involves the use of radioactive material [40].

#### Detection of autoantibodies against ENA

##### Gel precipitation assays

Techniques of precipitating antibodies to ENA were discovered and used as diagnostic tools in CTD almost 5 decades ago [40]. The early work relied mainly on gel based techniques i.e. double immunodiffusion (DID) or counter current immunoelectrophoresis (CIE)) [41,42]. CIE has been shown in several studies to be more sensitive than DID [35,43]. These gel precipitation assays however have some limitations. They are not quantitative and disease sensitivity is poor [31]. Therefore several other approaches were explored, with the aim of increasing assay sensitivity but without a loss of disease specificity.

##### Passive haemagglutination (PHA)

The PHA method was quite popular in the late 1970s but has since been superseded by EIA/ELISA and western blot. Analysis appears to have been restricted to anti-Sm and anti-RNP antibodies. Although assay sensitivity is high, some problems with specificity, in particular with differentiating anti-Sm from anti-U1-RNP, have been described [43].

##### Western (immuno) blot

Immunoblotting was introduced in the 1980s and has been useful in refining our understanding of the spectrum of ANA. In this method first the nuclear and cytoplasmic antigens are separated according to their molecular weight by polyacrylamide gel electrophoresis and then transferred onto a membrane or strips. Antigen containing strips are incubated with control or patient serum. If present in the serum, a particular ANA binds to the specific antigen on the strip. After repeated washing and incubations with two types of conjugates and a chromogen substrate, positive reactions are indicated by a band on the strip. The specificity of the antibody is defined by the identification of the positive bands in comparison with the positive control strip. Although considered to be highly sensitive for anti-ENA a major disadvantage with this technique is that antibodies directed against conformational epitopes are not detected [44]. In several studies, immunoblotting was found to be particularly insensitive for anti-SSA/Ro [34,45]. This apparent paradox is explained by the fact that the higher resolution seen in western blotting detects only linear epitopes whereas some 15% of anti-SSA/Ro antibodies react only with conformational epitopes not detectable in western blot. Moreover western blot is considered to be inadequate for anti-Scl70 with an assay sensitivity of only 25% [35]. Also sometimes there may be a problem with the under detection of U1-RNP [46,47]. Again, disease specificity is poor and in studies on normal populations false positives are not infrequent.

##### Dot blot

The dot blot method is a qualitative assay, which utilizes strips of nitrocellulose on which purified antigens are blotted at pre-located spots. The antigen sources used are bovine and rabbit thymus (for SSA, Sm and Scl-70) or calf spleen and rabbit thymus (for SSB and Sm/RNP). The strips are incubated with a 50-fold dilution of patient serum followed by incubation with an alkaline phosphatase-protein A conjugate. Finally the test strips are stained with 5-bromo-4-chloro-3-indolylphosphate/nitroblue tetrazolium. Positive strips are stained as a blue spot [48]. The dot blot test is advantageous for time management as the test requires just 30 minutes, can be easily performed and relatively cheaper. A major drawback however is the blotting of RNP antigen in combination with Sm antigen. This implies that if both the Sm spot and the Sm/RNP spot are positive the presence of Sm antibodies alone cannot be distinguished from the combined presence of Sm and RNP antibodies.

##### Line blot Immunoassay

Line blot Immunoassay is another qualitative test which reveals antibody reactivity to antigens that are applied as distinct lines on a membrane. Specific nuclear antigens are applied to nitrocellulose strips at equal distances. The required number of strips is placed to the respective row of the incubation tray. To rehydrate and to block free binding sites against unspecific binding, the strips are incubated with buffer, containing blocking protein. After discarding the blocking buffer, the membrane strips are incubated with prediluted serum samples. According to their specificity, autoantibodies, if present in the sample bind to the antigens are traced by alkaline phosphatase conjugated anti-human-IgG antibodies and appear as blue stained bands on the strips. Like dot blot, line blot is also easy to use and requires less processing time and is comparable to ELISA in sensitivity and specificity. Automated interpretation is also possible [49].

##### Multiplex Immunoassay (MIA)

The newly developed MIA enables the detection of multiple specific ANA as separate entities at the same time [50-53]. In MIA the patient sera is incubated in a well containing a multiplexed mixture of the bead suspension. The bead suspension consists of polystyrene microspheres that are conjugated with different antigens and nuclear extract of Hep-2 cells. If the patient serum contains antibodies to any of the antigens or Hep-2 nuclear extract, the antibody will bind to the immobilized antigen on 1 or more of the bead sets. The antibody-antigen-bead complex is then incubated with phycoerythrin conjugated goat anti-human IgG and the bead suspension is then analyzed by the immunoassay analyzer. The beads are uniquely identified by their corresponding fluorescent dye, and the amount of phycoerythrin conjugate is determined for each antigen. Multiplex ANA testing is being claimed to be more efficient and technically less challenging than IF-ANA screening, decreases false positivity, removes subjectivity and is more efficient than conventional ELISA [51].

##### Flowcytometry

Flowcytometry with autoantigen-coated fluorescent beads has been gaining popularity in recent years. It gives quantitative results based on reactivity with a mixture of bead subsets that are each labeled with a unique combination of internal fluorescent signal and antigen. Fluorescent beads-based techniques, also commonly referred to as Reflex ANA, are claimed to have multiple advantages such as simultaneous testing for recognition of several antigens, automation, cost effectiveness and high sensitivity. However, most significant limitation of this method is that it provides only a single result for each analysis. Often, multiple tests are necessary in order to be able to report a complete repertoire of required autoantibody results [54,55].

##### Antigen microarray

Antigen microarray currently not widely performed but may be an excellent advancement for simultaneous measurement of multiple ANA. This is a nanotechnology technique in which pre-synthesized antigens are printed on polystyrene and incubated with serum samples and then with horseradish peroxidase-conjugated secondary antibodies and chemiluminescent substrates. Light signals produced are captured by a charge-coupled device camera based chip reader. Antibodies are quantified by use of calibration curves [56]. The method offers the advantages of complete automation, consistent performance, cost-effectiveness and more precise measurement of antibody levels. The results are largely comparable to those obtained with techniques currently used in clinical laboratories [57-59]. Microarray may also be suitable for the discovery and evaluation of novel autoantibodies [60].

Among the above mentioned techniques choice depends on multiple factors i.e. test required for screening or detection of specific ANA, sensitivity and specificity of the test, availability, cost effectiveness, time taken and skill required to perform the test. Advantages and disadvantages for each of these tests have been compared in table 4.

**Table 4:** Performance of various tests used for detection of specific antibodies

**Method**	**Advantages**	**Disadvantages**
**IF-ANA**	Cost effective Easy to perform High sensitivity and specificity	Time consuming Can give false positive results ENA categorization difficult Requires trained personnel
**ELISA**	Automated Potential for quantification High sensitivity Potential for antibody class definition	Potential for false positives Expensive Requires purified antigen
**DID**	Cost effective High specificity Detects multiple antibodies at a time	Low sensitivity Subjective interpretation Need for large volumes of prototype sera
**CIE**	Cost effective High specificity Detects multiple antibodies at a time Faster than double diffusion	Modest sensitivity Subjective interpretation Need for large volumes of prototype sera
**PHA**	Semiquantitative High specificity	Time consuming Needs purified antigen
**Western blot**	More sensitive than DID and CIE High specificity	Expensive Time consuming Detects linear epitopes only
**Dot/Line blot**	Easy to perform, rapid High sensitivity and specificity Automation possible	Qualitative Distinction between certain antibodies difficult
**MIA**	Detects multiple antibodies at a time Quantitation possible	Expensive
**Flowcytometry**	Cost effective Automated High sensitivity	Provides single result at a time
**Microarray**	Detects multiple antibodies at a time Complete automation possible High sensitivity and specificity Cost effective	Not widely available

## Guidelines for detection of ANA

A positive ANA result in conjunction with clinical findings is diagnostic therefore frequently asked by the clinician in case of suspected CTD. Since different ANA are associated with one or other CTD a systematic approach has to be followed while performing these tests. Therefore initially screening is carried out usually by IF-ANA/ELISA and if positive more specific tests are performed based on clinical findings and IF-ANA staining patterns (table 3).

Autoantibody to dsDNA is specific and diagnostic for SLE and levels are elevated during active disease. Therefore in a case of suspected SLE if homogenous pattern is observed on IF-ANA further tests i.e. CLIF, ELISA, blotting tests etc. may be done to confirm dsDNA. Similarly anti-Sm is highly specific for SLE and needs confirmation by other tests i.e. Blotting etc. but is present in only 10% of SLE cases.

Anti-SSA/Ro antibody although more common in Sjogren's syndrome but can also be found in 30% cases of SLE with cutaneous involvement. Therefore if IF-ANA shows speckled/peripheral pattern further tests i.e. Blotting, MIA are required for detection of anti-SSA/Ro antibody. Clinical significance and detection methods for anti-SSB/La are similar to that for anti-SSA/Ro except that it is less common and may indicate minor course of disease. While presence of these two autoantibodies supports Sjogren's syndrome they are not much needed for diagnosis. Anti-Scl-70 autoantibody found in scleroderma (SS) gives a fine speckled staining pattern on IF-ANA and can be confirmed by immunodiffusion techniques but its detection is also not a necessity for diagnosis.

Antinucleolar antibodies are a group of autoantibodies which give nucleolar staining pattern. Most common of these are anti-PM-Scl, anti-RNA polymerase I-III and anti-U3-RNP (antifibrillarin). Although seen in scleroderma and polymyositis (PM) their detection is also not widely practiced [24].

**Figure 2:**
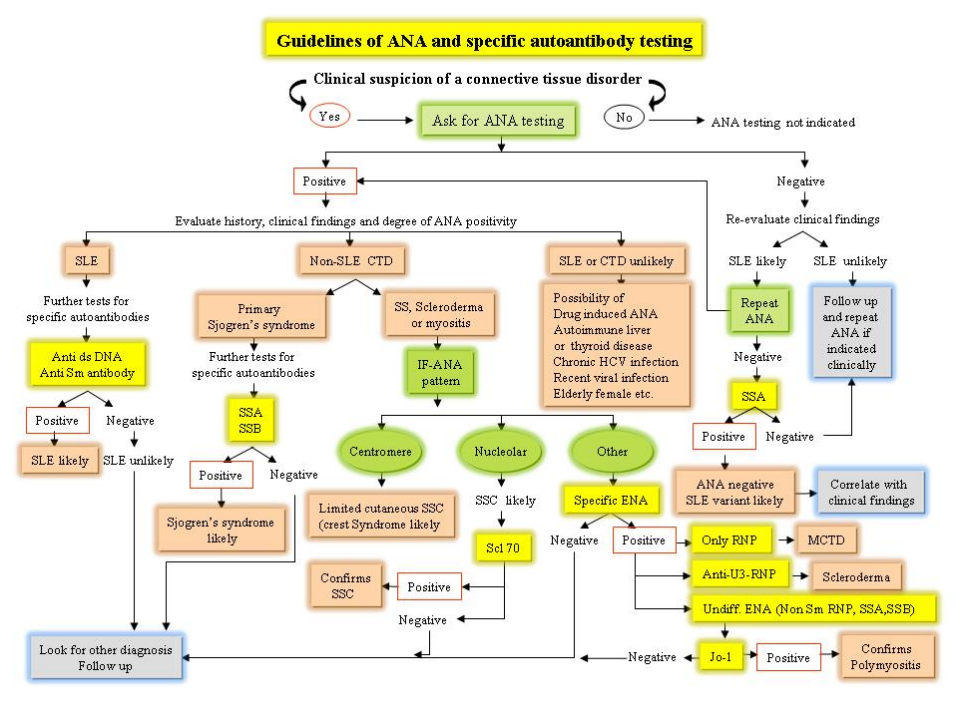
**Algorithmic approach for ANA testing**.

A protocol generally followed by the clinicians and step by step approach to detect all these autoantibodies has been described in figure 2. A summary of certain other guidelines [24,61] to be considered are:

- ANA testing is not helpful in confirming a diagnosis of rheumatoid arthritis or osteoarthritis therefore should not be used in such conditions.

- ANA testing is not recommended to evaluate fatigue, back pain or other musculoskeletal pain unless accompanied by one or more of the clinical features in favor of a CTD.

- ANA testing should usually be ordered only once.

- Positive ANA tests do not need to be repeated.

- Negative tests need to be repeated only if there is a strong suspicion of an evolving CTD or a change in the patient's illness suggesting the diagnosis should be revised.

- A positive ANA test is important only in conjunction with clinical evaluation and in the absence of symptoms and signs of a CTD; a positive ANA test only confounds the diagnosis. A positive ANA test can also be seen in healthy individuals, particularly the elderly or in a wide range of diseases other than CTD, where it has no diagnostic or prognostic value.

Recommendations in the guidelines may further evolve over time, as newer analytic methods and additional clinical research yield important results.

## In future!

The future for ANA detection looks very promising. We have come a long way from the simplest test like LE cell method to fully automated ELISAs to nanotechnology. Future development will undoubtedly include more sophisticated instrumentation with ultra sensitive detection, faster turnaround time, and increased throughput in ANA detection. Advances in the new technologies like multiplex immunoassays and antigen microarrays offer an attractive alternative to traditional ELISA, immunoblot, and IFA techniques. Rapid development in the area of quantum dots and other fluorescent nanoparticles will also eventually benefit routine clinical laboratory analysis.

## Competing interests

The authors declare that they have no competing interests.

## Authors' contributions

YK is primarily responsible for design of the study, literature search and drafting of the manuscript, AB and RWM participated in the sequence alignment and made critical revision for important intellectual content. All authors read and approved the final manuscript.
